# Non-anesthesiologist-administered propofol sedation for gastrointestinal endoscopy in ASA III patients: Eight-year experience

**DOI:** 10.1055/a-2857-9602

**Published:** 2026-05-12

**Authors:** Giuliano F. Bonura, Pablo Cortegoso Valdivia, Gabriella Frassanito, Noemi Gualandi, Paola Soriani, Federica Indulti, Valentina Zadro, Arianna Parrella, Tommaso Gabbani, Antonio Facciorusso, Konstantinos Triantafyllou, Mauro Manno

**Affiliations:** 1 Gastroenterology and Digestive Endoscopy Unit 18067 Azienda USL ModenaModenaItaly; 2 Gastroenterology and Endoscopy Unit 18630 University Hospital of ParmaParmaItaly; 3 Department of Clinical Research 6174 University of Southern DenmarkOdenseDenmark; 4 Surgical Research Unit 11286 Odense University HospitalOdenseDenmark; 5 Department Experimental Medicine 18976 Università del SalentoLecceItaly; 6 Second Department of Gastroenterology, Attikon University General Hospital 68993 National and Kapodistrian University of AthensAthensGreece

**Keywords:** Quality and logistical aspects, Sedation and monitoring, Training, Quality management

## Abstract

**Background and study aims:**

Sedation in gastrointestinal endoscopy is widely recognized for improving patient comfort, procedure quality, and endoscopist satisfaction. Although non-anesthesiologist-administered sedation (NAS) with propofol is well established for low-risk patients (i.e. American Society of Anesthesiologist [ASA] I-II), evidence for its safety in higher-risk populations remains limited. The aim of this study was to evaluate safety of NAS with propofol in ASA III patients undergoing diagnostic gastrointestinal endoscopy.

**Patients and methods:**

This observational study included ASA III patients who underwent elective gastroscopy or colonoscopy under NAS with propofol, with or without adjunctive midazolam and/or fentanyl, between 2017 and 2024. Sedation was administered by trained endoscopy personnel according to European Society of Gastrointestinal Endoscopy/European Society of Gastroenterology and Endoscopy Nurses and Associates guidelines. Anesthesia-related adverse events (ARAEs) were categorized as minor, moderate, or severe based on World Society of Intravenous Anesthesia definitions. Secondary outcomes included identification of potential risk factors for ARAEs.

**Results:**

Among 1423 procedures (43.4% gastroscopies, 56.6% colonoscopies), overall incidence of ARAEs was 5.2% (3.9% minor, 0.9% moderate, 0.4% severe), with no tracheal intubations or deaths. Hypotension (3.6%) was the most frequent and all such events were transient and reversible. Severe ARAEs were all bradycardia, successfully treated with atropine administration. The ARAE rate was higher with propofol + midazolam (8.6%) than with propofol + fentanyl (3.7%, *P* = 0.04). Logistic regression analysis identified lower body mass index as the only independent predictor of ARAEs ( *P* < 0.001).

**Conclusions:**

NAS with propofol appears safe and feasible in ASA III patients when performed by trained staff following standardized protocols and monitoring. These findings support expanding NAS with propofol to higher-risk populations, optimizing resources and access to endoscopic care.

## Introduction

Sedation in gastrointestinal endoscopy is widely recognized for improving patient comfort, procedure quality, and endoscopist satisfaction 1 2 . International guidelines recommend that sedation be offered routinely during both diagnostic and therapeutic endoscopic procedures 3 4 5 . Traditional sedation regimens often rely on benzodiazepine-opioid combinations, whereas propofol has become increasingly preferred due to its rapid onset, predictable titration, and short recovery times 6 7 . These advantages enhance patient comfort and procedure efficiency, potentially leading to higher detection and completion rates 1 8 .

Non-anesthesiologist-administered sedation (NAS) has been shown to be safe and effective in low-risk patients, that is, American Society of Anesthesiologists (ASA) I-II, with complication rates comparable to anesthesiologist-administered sedation 9 10 11 . Evidence specifically for NAS with propofol in this population also suggests a favorable safety profile when proper training and monitoring are in place 10 11 12 . Despite this, regulatory restrictions in many countries still limit propofol use to anesthesiologists, highlighting a gap between guideline recommendations and real-world practice 9 13 .

Safety of NAS with propofol in higher-risk populations, particularly ASA III patients, remains uncertain. These patients are more vulnerable to cardiopulmonary complications and available data are scanty. Observational studies suggest that NAS with propofol may be safe in this population 14 15 , but findings are often underpowered and heterogeneous, and expert reviews call for further investigation 16 17 . The aim of this study was to evaluate safety of NAS with propofol in ASA III patients undergoing gastrointestinal endoscopy.

## Patients and methods

### Study design

This was an observational, retrospective study conducted in a community hospital. The study was approved by the Research & Development oﬃce of our hospital, reported according to STROBE guidelines 18 and conducted in accordance with the declaration of Helsinki.

### Study population

Patient records were eligible for inclusion if they referred to adults (aged 18 years or older) who underwent diagnostic gastroscopy or colonoscopy under NAS with propofol. Only individuals classified as ASA physical status III with documented informed consents for both the endoscopic procedure and sedation were included. The ASA classification for each patient was systematically reviewed and confirmed in consultation with an anesthesiologist.

Exclusion criteria included records that referred to patients younger than 18 years, those classified as ASA I, II, IV, V, pregnant or breastfeeding women, and individuals unable to provide informed consent. Patients who underwent procedures with anesthesiologist-directed sedation or performed in an urgent or emergency setting were also excluded.

### Procedure

Sedation was administered by a member of the endoscopy team (endoscopist or nurse) who had completed the European Society of Gastrointestinal Endoscopy (ESGE)/European Society of Gastroenterology and Endoscopy Nurses and Associates (ESGENA) curriculum for sedation in gastrointestinal endoscopy and was exclusively dedicated to sedation during the procedure 19 .

Each patient underwent a pre-procedure assessment including medical history, comorbidities, allergy, medications, ASA classification, and body mass index (BMI). BMI was categorized as follows: normal weight (< 25 kg/m ^2^ ), overweight (25–29.9 kg/m ^2^ ), obesity class I (30–34.9 kg/m ^2^ ), obesity class II (35–39.9 kg/m ^2^ ), and obesity class III (≥ 40 kg/m ^2^ ).

Sedation protocols were established in collaboration with anesthesiologists. Propofol was administered as intermittent boluses, with or without adjunctive midazolam and/or fentanyl, to achieve deep sedation as assessed by the Richmond Agitation Sedation Scale. Protocol dosages were as follows: propofol 0.3 to 0.6 mg/kg (with an additional dose of 0.3–0.4 mg/kg after 2 minutes), preceded or not by midazolam 0.02 to 0.03 mg/kg and/or fentanyl 0.6 to 1.3 μg/kg.

All patients received supplementary oxygen (4 L/min) and multiparametric monitoring, including continuous pulse oximetry, continuous electrocardiography, heart rate monitoring, and noninvasive arterial blood pressure measurement every 5 minutes, as recommended by European guidelines 4 .

Following the procedure, all patients were transferred to the recovery room, where they were continuously monitored until full recovery of baseline clinical status, including stable vital signs, adequate spontaneous ventilation, and regained consciousness.

### Primary outcome

The primary outcome was safety of NAS with propofol in ASA III patients, assessed according to consensus definitions of anesthesia-related adverse events (ARAEs) established by the International Sedation Task Force of the World Society of Intravenous Anesthesia 20 .

Severe ARAEs were oxygen saturation < 75%, oxygen saturation < 90% for > 60 seconds, prolonged apnea (> 60 seconds), cardiovascular collapse/shock, cardiac arrest (absent pulse), need for tracheal intubation, administration of neuromuscular blockers, vasopressors (including epinephrine or atropine), permanent neurological deficit, pulmonary aspiration syndrome, or death.

Moderate ARAEs were oxygen saturation 75% to 90% for < 60 seconds requiring bag-valve mask-assisted ventilation and/or oral/nasal airway insertion, changes in vital signs > 25% from baseline requiring treatment (e.g., hypotension treated with IV fluids), or unplanned hospitalization/escalation of care (transfer to intensive care unit or prolonged hospitalization).

Minor ARAEs were oxygen saturation 75% to 90% for < 60 seconds corrected with airway repositioning and/or increased oxygen flow, transient vital sign changes (> 25% from baseline) requiring no therapy, and no adverse outcome.

Patients with airway obstruction (oxygen desaturation > 90%) were managed with chin lift or jaw thrust maneuvers by the dedicated member of the endoscopic team. These transient events were not recorded, in accordance with standardized definitions.

### Secondary outcomes

Secondary outcomes included identification of potential risk factors for ARAEs. Variables analyzed were patient age, sex, BMI, comorbidities, procedure time, and procedure type. In addition, we evaluated whether sedation regimens, according to the sedatives administered, were associated with ARAEs.

### Statistical analysis

Categorical variables were presented as numbers and percentages, whereas continuous variables were expressed as median and interquartile range (IQR) based on their distribution, which was assessed using the Shapiro-Wilk test. For the primary outcome, incidence of ARAEs was reported as total numbers and percentages with 95% confidence intervals (CIs).

Incidence of ARAEs was compared across different sedation regimens (propofol alone, propofol+midazolam, propofol+fentanyl, and propofol+fentanyl+midazolam) using the χ ^2^ test, with post-hoc pairwise comparisons performed using Bonferroni correction.

To identify potential predictors for the secondary outcome, univariable logistic regression analysis was performed for each variable of interest. A backward stepwise selection procedure was applied to obtain the final model, including only independent predictors with *P* < 0.05. To explore findings from the final model, secondary analyses were conducted, including an analysis of variance (ANOVA) to compare mean per-kilogram dosages of propofol, fentanyl and midazolam across different BMI classes.

All analyses were performed using R software (version 4.5.1; R Foundation for Statistical Computing, Vienna, Austria).

## Results

**Fig. 1:**
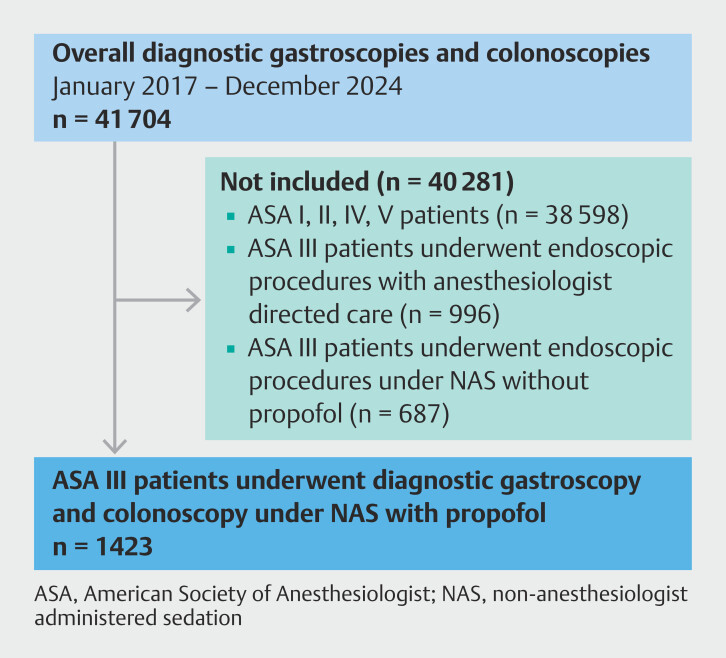
Flowchart of eligibility.

Between January 2017 and December 2024, a total of 1219 ASA III patients underwent 1423 endoscopic procedures under NAS with propofol ± adjunctive midazolam and/or fentanyl of 41704 diagnostic gastroscopies and colonoscopies performed in our unit during the study period, comprising 618 gastroscopies (43.4%) and 805 colonoscopies (56.6%) ( Fig. 1 ).

Four distinct sedation regimens were administered: propofol alone in 19.3% of cases (274/1423), propofol+midazolam in 18.7% (266/1423), propofol+fentanyl in 32.1% (458/1423), and propofol combined with both fentanyl and midazolam in 29.9% (425/1423).

A total of 79.2% of patients (965/1219) were aged 65 years or older.

Baseline patient and procedure characteristics are presented in Table 1 .

**Table:** **Table 1** Baseline patient and procedure characteristics.

Patient characteristics (n = 1219)	
Males, n (%)	682 (55.9)
Age	
Median [IQR]	75.2 [67.0–81.2]
> 65 years, n (%)	965 (79.2)
BMI, median [IQR]	26.6 [23.4–29.4]
BMI Distribution, n (%)	
Normal weight (< 25)	446 (36.6%)
Overweight (25–29.9)	506 (41.5%)
Obesity class I (30–34.9)	163 (13.4%)
Obesity class II (35–39.9)	67 (5.5%)
Obesity class III (≥40)	37 (3.0%)
Comorbidities	
Cardiovascular	906 (74.3)
Diabetes	301 (24.7)
Pulmonary	146 (12.0)
Neurological	126 (10.3)
Renal	119 (9.8)
Hepatic	78 (6.4)
**Procedure characteristics (n = 1423)**	
Type, n (%)	
EGD	618 (43.4)
Colonoscopy	805 (56.6)
Duration, median [IQR]	
EGD (minutes)	12 [8–15]
Colonoscopy (minutes)	23 [16–32]
BMI, body mass index; EGD, esophagogastroduodenoscopy; IQR, interquartile range.	

### Primary outcomes

Overall, ARAEs were recorded in 5.2% of cases (74/1423), of which 3.9% (55/1423) were classified as minor, 0.9% (13/1423) as moderate, and 0.4% as severe (6/1423).

Incidence of ARAEs was 4.6% for colonoscopy and 6.0% for upper endoscopy, with no statistically significant difference between the two groups (χ ^2^ test, *P* = 0.29).

Specifically, hypotension was the most frequent event, reported in 3.6% of cases (52/1423) and requiring no medical intervention, whereas 0.8% of cases of hypotension (12/1423) were successfully treated with intravenous (IV) fluids.

Minor desaturation occurred in 0.2% of cases (3/1423) and was managed with chin lift or jaw thrust maneuvers by the dedicated nurse and/or increased oxygen flow. A single case (0.07%) of moderate desaturation required transient bag-valve mask-assisted ventilation.

Atropine was successfully administered in six cases of 1423 patients (0.4%) for bradycardia, without any consequences for the patients. No other severe ARAEs occurred, particularly no need for tracheal intubation or death occurred.

A summary of ARAEs is presented in Table 2 .

**Table:** **Table 2** Distribution and severity of anesthesia-related adverse events

	Overall (n = 1423)	EGD (n = 618)	Colonoscopy (n = 805)	*P* value
**Overall** , n (%)	74 (5.2)	37 (6.0)	37 (4.6)	0.290
**Major** , n (%)				
Bradycardia	6 (0.4)	2 (0.3)	4 (0.5)	
**Moderate** , n (%)				
Hypotension	12 (0.8)	7 (1.1)	5 (0.6)	
Desaturation	1 (0.1)		1 (0.1)	
**Minor, n (%)**				
Hypotension	52 (3.6)	27 (4.4)	25 (3.1)	
Desaturation	3 (0.2)	1 (0.2)	2 (0.3)	
EGD, esophagogastroduodenoscopy				

### Secondary outcomes

**Fig. 2:**
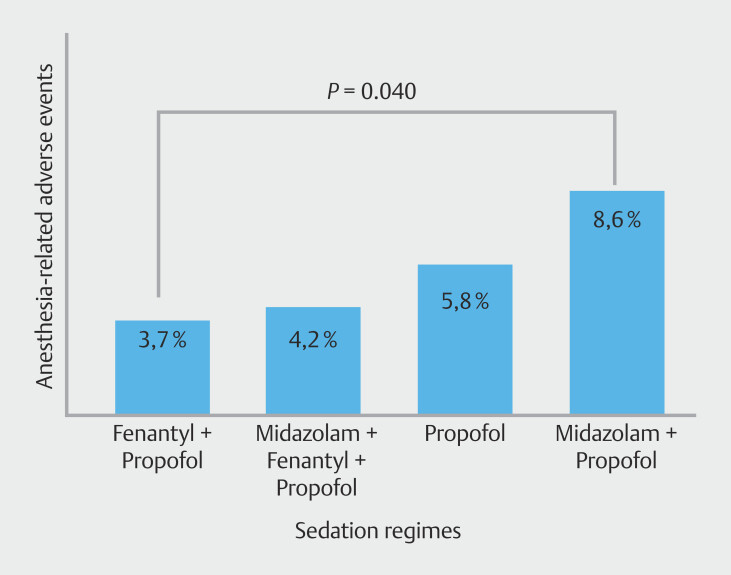
Incidence of anesthesia-related adverse events by sedation regimen.

A significant difference in incidence of ARAEs was observed among the four sedation regimens (χ ^2^ test, *P* = 0.023). The highest incidence of ARAEs was recorded in the propofol+midazolam group (8.6%, 23/266), followed by propofol alone (5.8%, 16/274). Conversely, regimens including fentanyl were associated with a lower incidence of ARAEs, specifically the propofol+fentanyl+midazolam group (4.2%, 18/425) and the propofol+fentanyl group (3.7%, 17/458). Post-hoc pairwise comparisons revealed a statistically significant difference ( *P* = 0.04, OR 2.46) between the highest-risk group (propofol+midazolam) and the lowest-risk group (propofol+fentanyl) ( Fig. 2 ).

**Fig. 3:**
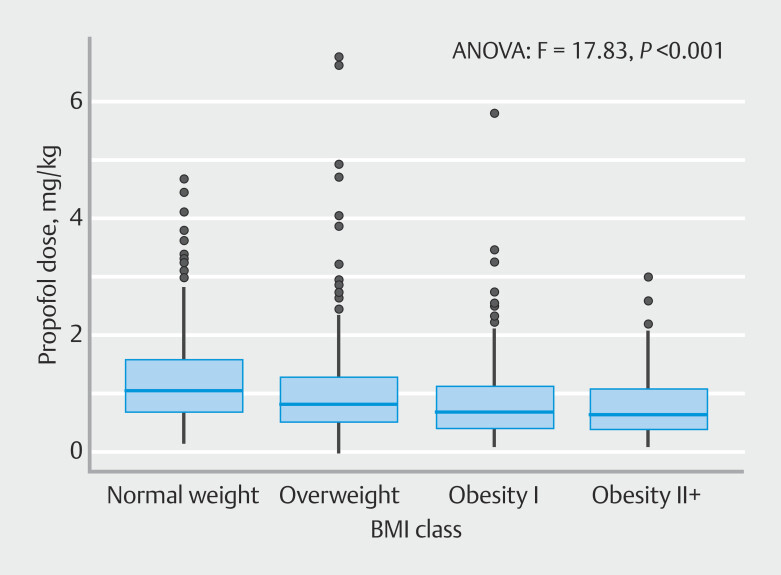
Comparison of per-kilogram propofol dosage across body mass index classes.

In the final univariate logistic regression model, only one factor emerged as an independent and statistically significant predictor of ARAEs: lower BMI (OR 0.92, 95% CI 0.87–0.96, *P* < 0.001) ( Table 3 ). Variables related to procedure type and duration were not retained in the final model after the backward selection process. Regarding severe ARAEs, the extremely low incidence (0.4%, 6/1423) precluded formal regression analysis. Descriptively, five of these six patients had preexisting cardiac comorbidities, but no reliable risk patterns could be identified due to the small sample size. Exploratory analyses to investigate these findings revealed a statistically significant negative correlation between BMI and administered per-kilogram propofol dose (r = –0.196, *P* < 0.001). An ANOVA test confirmed that patients in higher BMI classes (overweight and obese) received significantly lower mean per-kilogram propofol dosages compared to the normal-weight group ( *P* < 0.001) ( Fig. 3 ). No differences were observed in the mean per-kilogram fentanyl and midazolam dosages.

**Table:** **Table 3** Univariable logistic regression analysis of independent predictors for ARAEs.

	Univariable analysis	
Predictors	OR (95% CI)	*P* value
Age	0.99 (0.97–1.02)	0.579
Sex	1.07 (0.67–1.72)	0.794
BMI	0.92 (0.87–0.96)	< 0.001
Comorbidity (nephropathy)	1.52 (0.77–2.79)	0.197
Comorbidity (diabetes)	0.78 (0.43–1.33)	0.379
Comorbidity (cardiopathy)	1.17 (0.69–2.10)	0.579
Comorbidity (pneumopathy)	1.11 (0.53–2.10)	0.774
Comorbidity (hepatopathy)	1.03 (0.35–2.37)	0.957
Comorbidity (neuropathy)	1.04 (0.45–2.08)	0.922
Procedure duration (min)	0.99 (0.97–1.01)	0.327
Procedure type (EGD)	1.32 (0.83–2.12)	0.243
ARAE, anesthesia-related adverse event; BMI, body mass index; CI, confidence interval; EGD, esophagogastroduodenoscopy; OR, odds ratio.		

## Discussion

This observational study evaluated safety of NAS with propofol in ASA III patients undergoing routine gastrointestinal endoscopy. In a large cohort of 1423 of 41,704 diagnostic gastroscopies and colonoscopies performed over 8 years, overall incidence of ARAEs was very low (5.2%), with severe ARAEs occurring in only 0.4% of cases and no cases of tracheal intubation or death.

A key finding of this study is that most ARAEs were minor and transient. Hypotension was the most frequent ARAE, generally transient and self-limiting, occurring in 3.6% of cases and typically requiring no intervention. Only 0.8% of cases required IV fluids and none required vasopressor support. Hypotension is a well-known effect of propofol administration and it is typically related to the higher cumulative dosages. However, it is not considered a harmful event and it usually resolves promptly once the drug is withheld 21 . None of the patients who experienced hypotension had any clinical consequences.

Minor oxygen desaturation occurred in a very small proportion of patients (0.2%). Only a single case (0.07%) of moderate desaturation required brief bag-valve mask ventilation. The very low incidence of desaturation events was likely related to presence of a dedicated, well-trained team member able to promptly recognize and manage early signs of hypoxemia.

Only 0.4% of cases involved severe ARAEs, all of which consisted of bradycardia episodes successfully treated with IV atropine. Continuous multiparametric monitoring during the endoscopic procedures allowed early detection of bradycardia and atropine (0.5–1 mg) was routinely administered in cases of heart rate < 40 bpm. These events were classified as severe according to the standardized criteria adopted for ARAE reporting. Although bradycardia can be related to sedation, particularly to propofol administration, it may also result from transient vagal stimulation or luminal overdistension during endoscopic maneuvers, both of which typically resolve upon removal of the stimulus. Finally, no adverse clinical consequences occurred following either the bradycardia episodes or atropine administration.

Interestingly, we encountered no severe ARAEs such as tracheal intubation or death, further reinforcing the evidence that, with appropriate patient selection, structured protocols, trained endoscopic team, and adherence to international guidelines, the risk profile of NAS with propofol in higher-risk populations may be comparable to that observed in lower-risk cohorts 9 11 .

Type (upper vs. lower endoscopy) and duration of procedure were not associated with ARAEs, in line with earlier evidence indicating that patient-related rather than procedure-related factors predominantly determine sedation risk, in the setting of routine diagnostic procedures 11 17 .

Importantly, the sedation regimen appeared to influence risk of ARAEs. Specifically, propofol+midazolam regimen was associated with the highest incidence, whereas propofol+fentanyl regime showed the lowest. These findings may reflect pharmacologic receptor synergy between midazolam and propofol. However, choice of sedation regimen was not driven by predefined criteria and depended on the combination of the three agents. Owing to the retrospective design of the study, the rationale underlying regimen selection could not be systematically assessed. Although both regimens have been demonstrated as safe, particularly in low-risk patients 10 12 , high-quality randomized trials comparing them, especially in ASA III patients, are lacking. The observed differences should be confirmed in future studies. Nevertheless, increasing clinical experience in recent years suggests that the combination of propofol and fentanyl may provide comparable efficacy with a lower incidence of adverse events (ARAEs) compared with other sedation protocols.

Regarding analysis of potential ARAEs predictors, our results identified a lower BMI as independent risk factor for ARAEs. Patients with normal BMI received higher per-kilogram dosages of propofol, increasing significantly the rate of ARAEs. Conversely, patients in higher BMI categories received significantly lower weight-adjusted dosages of propofol, indicating that individuals with higher BMI, who may be at increased risk of cardiorespiratory complications, should be managed with caution 15 . This finding reflects the clinical practice of titrating the dose toward lean or ideal body weight rather than total body weight in obese patients to avoid relative overdosing.

Finally, most patients were elderly, with no increase in ARAEs compared with younger patients, suggesting that propofol sedation is safe in ASA III patients, even in the elderly.

Implementation of the ESGE and ESGENA sedation training program in our unit has already shown the safety of NAS with propofol in ASA I-II patients, with very low ARAEs rates 12 . This study demonstrates that NAS with propofol is safe even in ASA III patients, who are traditionally considered at high risk, showing a significantly lower rate of ARAEs compared with previous reports 14 15 . The possibility of safely performing NAS with propofol in ASA III patients carries important clinical and organizational implications. A recent national Italian survey involving 14 centers identified patient intolerance and ASA III status as the main reasons for rescheduling endoscopic procedures under anesthesiologist-directed sedation 22 .

Notably, in the two centers routinely performing NAS with propofol, no diagnostic procedures required anesthesiologist supervision. Thus, adoption of NAS with propofol may reduce need for anesthesiologist involvement, optimize resource utilization, and potentially lower procedure costs 8 13 22 . Moreover, it could improve access to endoscopic procedures for patients who might otherwise experience delays due to limited anesthesia availability 22 .

Some limitations should be acknowledged. First, the retrospective design may underestimate minor or transient events not consistently recorded in medical charts, although standardized definitions were used and systematic review of ASA classification in consultation with anesthesiologists further supports reliability of patient risk stratification. Second, this is a single center study, which may limit generalizability, even though it was conducted by trained and skilled personnel in the field of gastrointestinal sedation. Third, reliance on ASA classification alone may be insufficient to adequately capture heterogeneity of ASA III patients, potentially affecting selection of candidates suitable for NAS with propofol. Fourth, absence of a control group of anesthesiologist-administered cases precludes direct comparison of safety outcomes. Finally, another limitation of this study is lack of patient-reported outcomes, such as pain scores and overall patient satisfaction at the end of the procedure.

## Conclusions

In conclusion, our study highlights that NAS with propofol for gastrointestinal endoscopic procedures can be safely administered in ASA III patients, when performed by appropriately trained personnel under standardized monitoring and protocols. Although these findings cannot be generalized to or recommended for all ASA III patients, they suggest potential feasibility of NAS with propofol in selected higher-risk populations and contribute to ongoing discussion about its possible use within regulated endoscopic practice. Further multicenter, prospective, and controlled studies are needed to better define risk factors and to refine patient selection and sedation strategies in this group.
